# Bone Metastasis From Glioblastoma Multiforme: A Case Report

**DOI:** 10.7759/cureus.25464

**Published:** 2022-05-29

**Authors:** Martin Zapata Laguado, Julian M Baez, Angela Luna, Carolina Mantilla, Maribel Palencia

**Affiliations:** 1 Oncology, Universidad El Bosque, Bogota, COL; 2 Oncology, Instituto Nacional de Cancerología, Bogota, COL; 3 Internal Medicine, Universidad Autónoma de Bucaramanga, Bucaramanga, COL; 4 Pathology, Instituto Nacional de Cancerología, Bogota, COL

**Keywords:** chemo radiotherapy (chemo-rt), glioma resection, extracranial metastases, bone metastasis, glioblastoma multiforme

## Abstract

Glioblastoma multiforme (GBM) is the most frequent neoplasm of the central nervous system (CNS) with a low incidence in people. The heterogeneous characteristics of this malignant tumor make the overall survival one of the shortest.

Metastatic lesions due to glioblastoma are mainly reported in liver, lungs and leptomeningeal spaces, existing in worldwide literature with very few reported cases. The osseous tissue continues to be an exceptional place to present metastases. This is the reason why we report one case of a young adult with extensive lytic compromise confirmed by histopathological features.

## Introduction

The glioblastoma multiforme is the most frequent and aggressive neoplasm of the central nervous system (CNS) with an incidence estimated at less than 10 per 100,000 people [1]. The heterogeneous characteristics of this malignant tumor make the overall survival one of the shortest, supported by anatomical issues such as the lack of lymphatic drainage and being surrounded by a hematoencephalic barrier, making distant metastases a rare condition [2]. Metastatic lesions due to glioblastoma are mainly reported in the liver, lungs, and leptomeningeal spaces. Only 200 cases have been reported in worldwide literature. The osseous tissue continues to be an exceptional place to present metastases [3]. It belongs to the grade IV of the world health organization (WHO) classification and represents nearly 60% of brain malignant tumors in adults [4,5]. The prognosis is poor, with a median overall survival between 15 to 18 months, and overall survival of five years (5%) [6,7].

The extracranial metastasis is unusual, the frequency is reported as less than 1% [8]. With the arrival of new therapies, it is important to recognize this unusual presentation that could appear in some patients.

Here, we report a case of a young woman with multiple osseous metastases without presenting progression in the CNS despite management with antiangiogenic therapy and alkylating therapy.

## Case presentation

**Figure 1:**
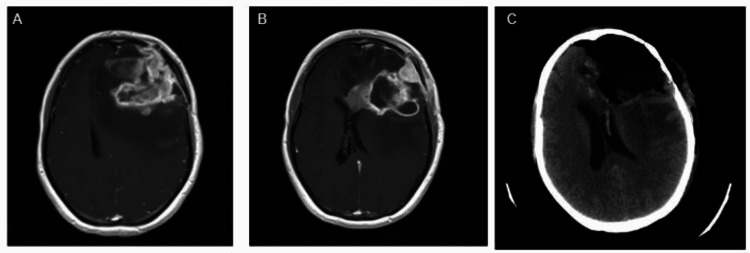
Images before and after surgical procedure A and B: Radiological evolution of glioblastoma consistent with progression during treatment (chemotherapy with temozolomide plus radiotherapy), C: Post-surgical changes of left frontal craniotomy and frontal lobectomy

A 32-year-old female, without past medical history presented with a headache located bitemporal with nausea and vomit, requiring magnetic resonance imaging. She was diagnosed with a GBM grade IV WHO classification, the size of the lesion was 53x54 mm, located at the inferior frontal gyrus with extension to the corpus callosum knee. At the time of the diagnosis, the performance status as per the Eastern Cooperative Oncology Group (ECOG) performance status scale was 1. Due to the extension of the tumor, as initial management, we started treatment as per the Stupp protocol i.e., chemotherapy based on alkylating therapy concomitant with radiotherapy consisting of a focal irradiation dose of 60 Gy in 2 Gy fraction with linear accelerators with nominal energy, and with tumor resection by the neurosurgery service as seen in Figure 1.

Two months after the culmination of chemoradiation therapy (during adjuvant treatment with temozolomide), the patient presented an unequivocal progression with a partial response in the primary target lesion. But having a leptomeningeal disease, the patient was treated with carmustine and bevacizumab. After four cycles of this therapy, the patient reached a complete response documented in an MRI of the brain.

**Figure 2:**
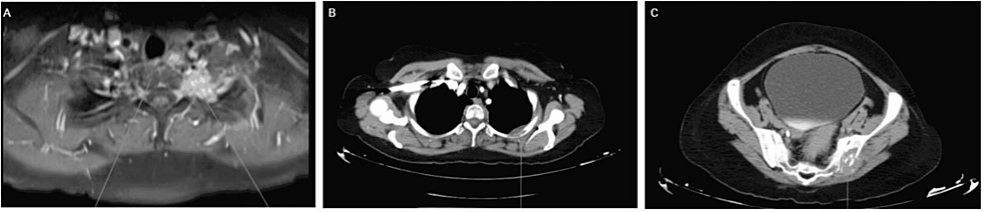
MRI and CT of metastatic lesions A: MRI of the hip - in the lower margin of the left interscalene triangle, a poorly defined mass can be seen that compromises the T1 and T2 roots;  B: CT of  the thorax - osteolytic lesion seen on the posterior aspect of the left third costal arch associated with an ill-defined oval soft tissue density mass of approximately 28 x 17 mm;  C: CT of abdomen and pelvis -  ill-defined osteolytic lesion seen in the left iliac flap with a soft tissue component of approximately 47 x 31 mm

**Figure 3:**
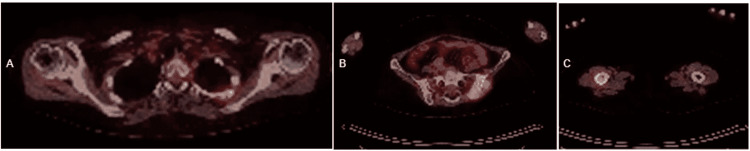
Hypermetabolic lesion on PET scan A: Hypermetabolic lesions located in the left transverse process of T1 are observed. The eft 3rd costal arch, left 4th costal arch, and the vertebral body of T2, are all seen with a tumor appearance;  B: Hypermetabolic lesions located in the left iliacus, left sacroiliac joint (standardized uptake value [SUV]max 13.6), seen with a tumor appearance;  C: Hypermetabolic lesion located in the middle third of the right femur with a tumor appearance PET: Positron emission tomography

On the sixth cycle of carmustine and bevacizumab, the patient started having pain in the hip and lower limbs. Therefore, an MRI of the hips and CT of the thorax, abdomen, and pelvis were taken (Figure 2). We documented a lytic compromise in the third rib arch, left posterior iliac region of 47x31 mm with an infiltrative bone marrow lesion in the middle third of the diaphysis of the femur with cortical anterior infiltration. After documenting that bone infiltration, we ordered a positron emission tomography (PET) that revealed bone disease in the sacroiliac joint and confirmed the disease in the previous images without revealing fractures (Figure 3). 

**Figure 4:**
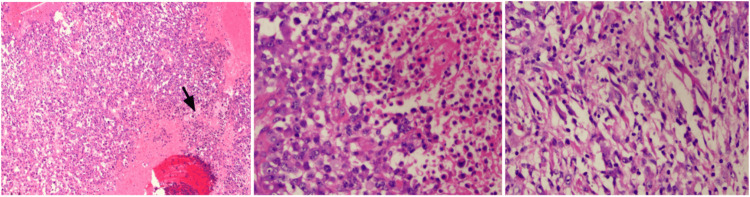
Biopsy results Left: Hematoxylin and eosin stain, 10x magnification. A proliferation of atypical astrocytes was seen with epithelioid and small cell morphology (arrow). Center: Hematoxylin and eosin staining, 40x magnification. In greater detail, the left half of the image corresponds to the morphology of the astrocytic tumor cell with an epithelioid appearance. Right: Hematoxylin and eosin staining, 40x magnification. Spindle cell differentiation.

**Figure 5:**
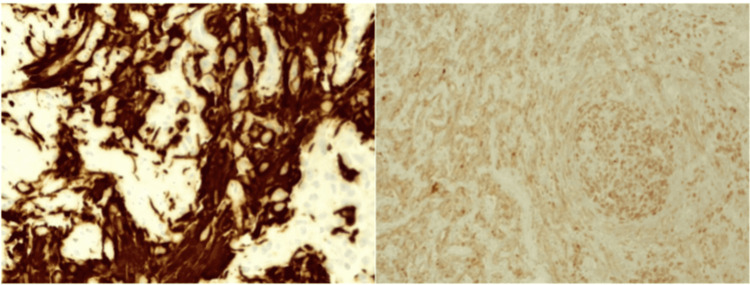
Left: Immunohistochemistry of glial fibrillary acidic protein (GFAP), located in the intermediate filaments of astrocytes. Right: Nuclear expression of protein gene product 9.5 (PGP 9.5), a member of the ubiquitin hydrolase family of proteins that is restricted to neural and neuroendocrine cells Tumor cells were negative for synaptophysin, Melan A, human melanoma black-45 (HMB45), and cytokeratin CAM 5.2. Cell proliferation index (Ki67) was 60% and phosphohistone H3 (pHH3) enhanced mitotic figures (up to 10 mitotic figures in 10 high-power fields). A second tissue sample sent as a biopsy of the left sacroiliac lesion showed metastatic involvement by a tumor with epithelioid and small cell characteristics similar to the images described, with positive tumor cells for protein gene product 9.5 (PGP 9.5) and negative for glial fibrillary acidic protein (GFAP), S100, SRY-box transcription factor 10 (SOX10), melanocyte inducing transcription factor (MITF). These markers confirm the cell lineage of the neoplasm [9].

Due to the rare incidence of metastatic disease in the context of glioblastoma, we performed a biopsy of the bone. The protein electrophoresis and immunofixation were normal. Despite the negative results, when the biopsy was repeated in the iliac aileron, it was reported as an epithelial appearance with an immunoprofile that favors primary origin in the CNS (Figures 4, 5). 

We ordered radiation therapy for the bone lesions, evaluation of BRAF mutations, and microsatellite instability. However, during the hospitalization, the patient presented with SARS-CoV-2 infection and developed viral pneumonia, and died due to this complication.

## Discussion

The glioblastoma multiforme is an aggressive neoplasm with a high intratumoral heterogeneity that confers therapeutic resistance. The unusual incidence of extracranial disease could be a multifactorial issue due to the low survival rate of the disease, the presence of a hematoencephalic barrier, and the lack of lymphatic drainage in the CNS [10]. 

For this reason, clinicians usually do not screen for extracerebral metastases. The National Comprehensive Cancer Network Guidelines do not recommend screening for metastases in patients with high-grade gliomas after maximal safe resection [11].

Young patients and healthier patients are more susceptible to developing extracranial metastases, likely due to a longer overall survival compared to elderly patients with multiple comorbidities. Regarding metastatic disease, there is a worse prognosis when it spreads to the lung and liver. Systemic metastases of the glioblastoma are found in 6% to 25% of the autopsies of patients with this affection [12], the presumable pathways producing dissemination of the disease could be perineural, lymphatic, vascular, contiguity, or iatrogenic. Being the most probable hematogenous malignancy despite the use of antiangiogenic therapy. The GBM cell invasion through the circulatory system or directly through the dura mater and the failure of the blood-brain barrier could favor GBM cell distribution through systemic circulation [13].

Surgery on the cerebral lesion has been related to hematogenous seeding of tumor cells but around 10% of the reported cases of extracranial glioblastoma diffusion occurred without surgical intervention and recent studies did not find this association between the procedure and tumor dissemination [12].

Hematogenous dissemination is increasingly found across cancers, including 21% of GBM patients. Thus, it is unclear why extracranial GBM is not seen with greater distant metastatic involvement. The possibility is that the reported incidence of GBM metastasis is underestimated, since the search for metastatic disease has not been protocolized and the short survival in this disease means that the risk of metastasis decreases, unlike those with oncological diseases with prolonged survival, which may have a higher risk to metastasize [14,15].

Extracranial bone metastases from GBM have been reported in the literature as single or multiple lesions [16]. This is a unique case because we observed a tumor relapse at the bone level and was confirmed with histopathology in a patient with a previous complete response at the CNS level. It is important for medical personnel, in general, to know the relevance of metastatic disease in these types of tumors since adjustments can be made regarding new treatment or follow-up in palliative units.

## Conclusions

Glioblastomas multiforme are aggressive tumors with short survival, which has been improved with current treatment regimens that involve wide surgical resection plus Stupp protocol. However, given the refractoriness of the treatment, there is little hopeful evidence for this pathology that allows an overall improvement in survival and where the heterogeneity of the tumor is the greatest impediment to a successful treatment. This is why the monitoring by the pain and palliative care team for symptomatic palliation and quality of life of patients must be based on multidisciplinary care for the risk of fatigue, seizures, mass effect, brain edema, radionecrosis, neurocognitive dysfunction, abnormalities of endocrine and psychiatric disorders, and venous thromboembolism.

Although we have found a greater number of reports of metastases in GBM, the pathophysiology of these lesions remains a mystery and the diagnosis remains exceptional. Our case report indicates the importance of recognizing and investigating metastatic disease since we can define prognosis and could open the door to the investigation of new targeted therapies to improve overall patient survival.

## References

[1] Hanif F, Muzaffar K, Perveen K, Malhi SM, Simjee ShU (2017). Glioblastoma multiforme: a review of its epidemiology and pathogenesis through clinical presentation and treatment. Asian Pac J Cancer Prev.

[2] Inda MM, Bonavia R, Seoane J (2014). Glioblastoma multiforme: a look inside its heterogeneous nature. Cancers (Basel).

[3] Zhang W, Cai YY, Wang XL (2021). Bone metastases of glioblastoma: a case report and review of the literature. Front Oncol.

[4] Castañeda CA, Casavilca S, Orrego E (2015). Glioblastoma: análisis molecular y sus implicancias clínicas. Rev Peru Med Exp Salud Publica.

[5] Trujillo FG, Noriega CC, Ramírez OJC (2014). Glioblastoma multiforme: actualidad en marcadores biomoleculares como factores de pronóstico a propósito de una serie de casos con sobrevida mayor a 2 años en el Instituto Nacional de Cancerología INC- Colombia. Acta Neurol Colomb.

[6] Schreck KC, Grossman SA, Pratilas CA (2019). BRAF mutations and the utility of RAF and MEK inhibitors in primary brain tumors. Cancers (Basel).

[7] McAleenan A, Kelly C, Spiga F (2021). Prognostic value of test(s) for O6-methylguanine-DNA methyltransferase (MGMT) promoter methylation for predicting overall survival in people with glioblastoma treated with temozolomide. Cochrane Database Syst Rev.

[8] Li Y, Yang S, Hao C (2020). Effect of BRAF/MEK inhibition on epithelioid glioblastoma with BRAFV600E mutation: a case report and review of the literature. Clin Lab.

[9] Campbell LK, Thomas JR, Lamps LW, Smoller BR, Folpe AL (2003). Protein gene product 9.5 (PGP 9.5) is not a specific marker of neural and nerve sheath tumors: an immunohistochemical study of 95 mesenchymal neoplasms. Mod Pathol.

[10] Engelhardt B, Carare RO, Bechmann I, Flügel A, Laman JD, Weller RO (2016). Vascular, glial, and lymphatic immune gateways of the central nervous system. Acta Neuropathol.

[11] Costa RB, Costa R, Kaplan J, Cruz MR, Shah H, Matsangou M, Carneiro B (2017). A rare case of glioblastoma multiforme with osseous metastases. Case Rep Oncol Med.

[12] Rossi J, Giaccherini L, Cavallieri F (2020). Extracranial metastases in secondary glioblastoma multiforme: a case report. BMC Neurol.

[13] Anghileri E, Castiglione M, Nunziata R (2016). Extraneural metastases in glioblastoma patients: two cases with YKL-40-positive glioblastomas and a meta-analysis of the literature. Neurosurg Rev.

[14] Forsyth TM, Bi WL, Abedalthagafi M, Dunn IF, Chiocca EA (2015). Extracranial growth of glioblastoma multiforme. J Clin Neurosci.

[15] Fonkem E, Lun M, Wong ET (2011). Rare phenomenon of extracranial metastasis of glioblastoma. J Clin Oncol.

[16] Fabi A, Vidiri A, Carapella C (2004). Bone metastasis from glioblastoma multiforme without central nervous system relapse: a case report. Anticancer Res.

